# Geochemical Evidence for the Evolution of the Deyang–Anyue Rift in the Ediacaran–Cambrian Period on the Northwestern Margin of the Yangtze Block

**DOI:** 10.1021/acsomega.6c03811

**Published:** 2026-09-14

**Authors:** Xiangyu Fan, Siqi Tang, Xiyan Yang, Xuan Liu, Cunhui Fan, Fei Huo, Ziyue Zhong, Rong Yang, Hu Li

**Affiliations:** † School of Geoscience and Technology, 74601 Southwest Petroleum University, Chengdu 610500, China; ‡ 124175 Sinopec Petroleum Exploration and Production Research Institute, Beijing 102206, China; § Sinopec Key Laboratory of Marine Oil and Gas Reservoir Development, Beijing 102206, China; ∥ National Energy Key Laboratory of Carbonate Oil and Gas, Beijing 102206, China; ⊥ Sichuan University of Science and Engineering, Yibin 644000, China

## Abstract

The evolution of a large intracratonic rift trough in the northern Sichuan Basin, China, along the northwestern margin of the Yangtze Block, is documented during the significant Ediacaran–Cambrian Transition (ECT). Three prevailing perspectives exist regarding the specific genesis of this rift: the inherited development of rift basins, erosional troughs, and tensional erosional troughs. However, the dynamic mechanisms underlying rift trough development and the precise timing of this transition remain contentious. Consequently, this paper conducts a systematic geochemical and chronological investigation for the first time, utilizing siliceous sediments from the Dengying Formation and detrital zircons from the Cambrian Canglangpu Formation. Detrital zircons are used to constrain the Neoproterozoic tectonic evolution of the northwestern Yangtze Block, and geochemical records of siliceous sediments are adopted to reconstruct the evolutionary history of the rift trough. The study’s results indicate the following: 1. Neoproterozoic zircons in the study area are dominated by continental arc signatures, with features of arc-associated orogenic compression and partially depleted mantle affinities. Crustal thickness variations calculated from zircon Eu/Eu* ratios indicate that arc-related extensional rifting occurred in the study area after ∼650 Ma. 2. The δ^30^Si values of chert range from −0.6‰ to 0.93‰, while those of siliceous dolostones vary between 0.66‰ and 3.26‰. These isotopic differences suggest that silica in chert originated from relatively deep-water environments with prominent biogenic sources, whereas silica in siliceous dolostones was derived from shallow marine settings through metasomatism. Trace element data reveal that both chert and siliceous dolostone experienced limited hydrothermal fluid modification during sedimentary and diagenetic stages. 3. During the ECT interval, crustal thinning drove the ascent of deep hydrothermal fluids and altered coeval seawater. Such weak hydrothermal overprint facilitated the preservation and deposition of bedded chert in deep-water conditions. Meanwhile, partial microbial dolostones underwent silicification metasomatism to form siliceous dolostones, and minor granular quartz silica filled pore spaces in microbial dolostones during late diagenesis.

## Graphical abstract

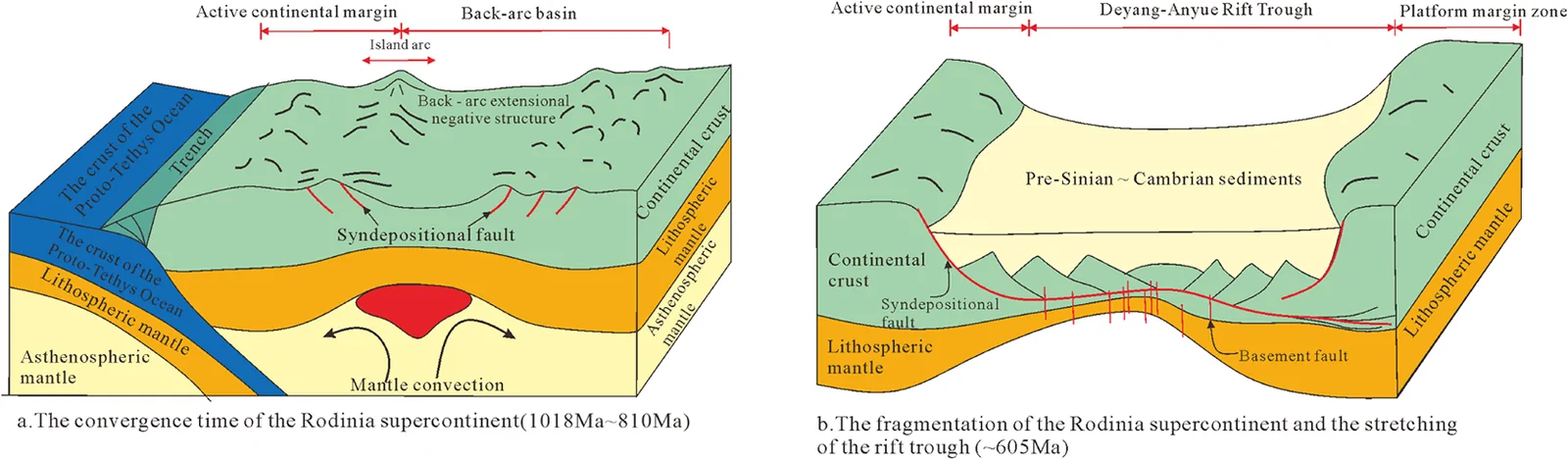

## 1 Introduction

The Ediacaran–Cambrian Transition (ECT) is one of the most important periods in the tectonic evolution of the Earth.^1–3^ A series of major geological events occurred during the ECT, which mainly include the following. 1. Abnormal fluctuations of many geochemical elements in the atmosphere and ocean (such as abnormal fluctuations of elements like carbon, iron, and silicon in seawater.^4–7^ 2. The transition of the global plate tectonic regime from the breakup of the Rodinia supercontinent to the aggregation of the Gondwana continent.^7–10^ 3. The rise and fall of the Ediacaran biota and the subsequent Cambrian Explosion of Life.^11,12^ The above important geological events indicate that the study of the geological records during the ECT can provide a basis for the geological transitions of the same period and also has great significance for oil and gas exploration.

Previous studies have shown that there is a large intraplate rift formed during the evolution of the Ediacaran–Cambrian Transition (ECT) period in the northern part of the Sichuan Basin, China, on the northwestern margin of the Yangtze Block (Figure 1a and Figure 1b). It is commonly referred to as the “Deyang–Anyue Rift”. The high-quality hydrocarbon source conditions within the rift trough have given rise to the Anyue Gas Field, which has the largest individual reserve scale in China.^3,13^ This demonstrates that the Dengying Formation of the Sinian System possesses tremendous natural gas exploration potential. Although the geological significance of hydrocarbons in the Deyang–Anyue Rift has been widely recognized, its formation time and genetic mechanisms have been debatable since its discovery.^13–17^ In the early stage of the study of the rift troughs, based on the limited seismic and drilling data, researchers proposed three hypotheses for its genesis: tension,^13,14^ erosion,^15^ and combined tension/erosion.^16–18^ With the continuous accumulation of drilling and field data in recent years, the understanding of the Deyang–Anyue Rift has been further improved. A series of outcrop studies on the northwestern outer margin of the Sichuan Basin showed that deep-water sediments were located in the Dengying Formation in the northern segment of the Deyang–Anyue Rift; these studies supported its sedimentary origin under an extensional setting.^5^ By comparing the abrupt changes in the sedimentary facies of the Dengying Formation between the sections inside the trough and the sections outside the trough, others indicated that the rift trough experienced synsedimentary extensional fault activity.^19^

**Figure 1:**
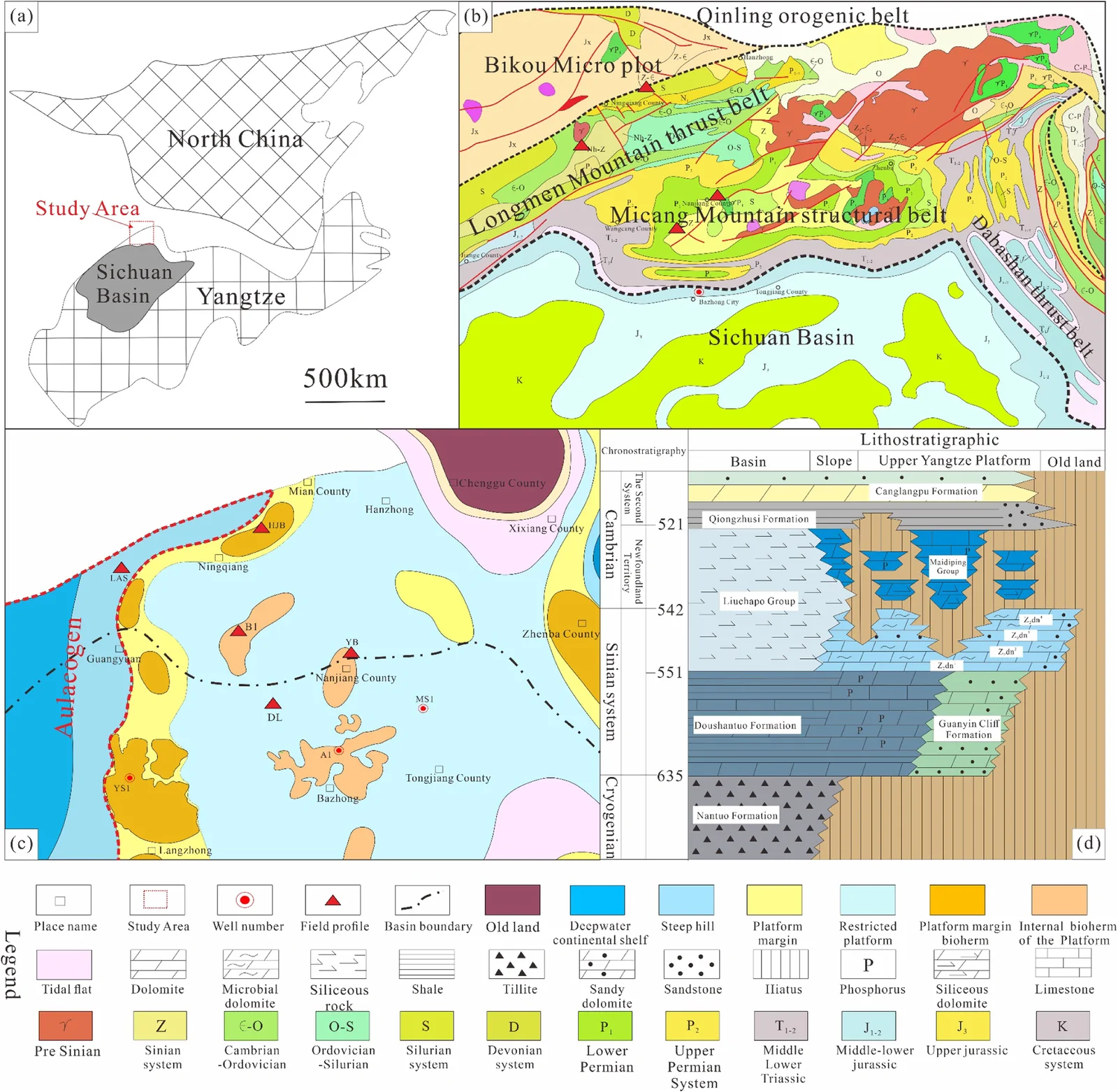
Geological Map of the northwestern Yangtze Block. (a) Structural location map of the research area; (b) geological map of the northwestern Yangtze Block; (c) lithofacies paleogeographic map of the northwestern Yangtze Block; (d) stratigraphic framework of the Cryogenian—Cambrian in the Yangtze Block.

Due to the geochemical characteristics of silicious sediments, silicious sediments can respond to the influences of volcanism, hydrothermal sedimentation, and terrigenous clastics, and identifying its impact on the paleoenvironment is important.^20–22^ The depositional period of the Dengying Formation in the northern Sichuan area was influenced by the Deyang-Anyue rift. The siliceous sediments were widely deposited in the trough, at the trough edge, and on the limited trough platform; these areas can be used for studies on the origin, tectonic background, and sedimentary evolution of the Deyang–Anyue Rift.

With respect to the formation mechanism of the rift trough, researchers have mostly conducted stratigraphic sequence and lithofacies analyses of the well section at the macro level, and detailed geochemical evidence is lacking. The samples in this study were collected from the strata surrounding the Deyang–Anyue rift trough, which have been investigated in previous studies. Although the genetic mechanism of this rift trough remains controversial, basic geological understanding has been established. On the basis of systematically summarizing diverse previous viewpoints on the formation mechanism of the rift trough, this study takes siliceous sediments from the Dengying Formation deposited during the evolutionary stage of the rift trough as a research medium. Combined with diverse silica-source characteristics, they provide more microscale geochemical evidence for the evolutionary process of the rift trough. Meanwhile, detrital zircon geochronology data from the Canglangpu Formation further supplement the macroscopic tectonic background during the evolution of the rift trough. Therefore, the authors utilized the geochemical characteristics of siliceous sediments during the ECT period and evidence from detrital zircon geochronology to explore the formation mechanism of the rift trough and the evolution of the tectonic setting, providing a new reference for the study of deep marine carbonate reservoirs in the Yangtze Block.

## 2 Geological Setting

The study area is located in the Guangyuan area on the northwestern margin of the Yangtze Block. Tectonically, it currently belongs to the Yangtze Continental Block and is mainly developed in the western segment of the collage zone between the Yangtze Block and the North China Plate (Figure 1a). It serves as a transitional area between the Sichuan Basin and the Qinling Orogenic Belt (Figure 1b).^23^ Although the specific mechanism remains debatable, a large number of studies suggest that the formation of the Deyang–Anyue Rift is related to the Rodinian continental breakup.^24^ The regional tensile motion accompanying this supercontinent breakup is likely the intrinsic driving force of trough formation. During this period, the North China Plate and the Yangtze Block rifted apart along the Qinling-Dabie Mountains belt to form an ocean basin, generating low-amplitude rifting within the Yangtze Block.^25^ During 750–635 Ma, the tension constantly weakened, and the rift basin gradually converted to a depressional basin. During the Sinian–Cambrian transitional period (635–541 Ma), the northern section of the Deyang–Anyue Rift had a flared geometry opening at the northwestern margin of the Yangtze Block and was connected to Guanghai.^5,26,27^ Until the depositional period of the Canglangpu Formation and Longwangmiao Formation in the early Cambrian, the Deyang–Anyue Rift was basically filled, and the sediment thickness and lithofacies inside and outside the trough were generally consistent.^13,28^ Against this tectonic background, the Sinian Dengying Formation is thick on both sides of the rift trough, and the overlying Lower Cambrian formation is thin or even absent; inside the rift trough, the thickness of the Dengying Formation decreases, whereas the thickness of the Lower Cambrian strata significantly increases. From west to east, the Dengying Formation in the study area represents a slope basin–carbonate platform sedimentary model (Figure 1c).

From bottom to top, the Sinian–Cambrian transitional strata in the study area are as follows: the Doushantuo Formation, the Dengying Formation, the Maidiping Formation, the Qiongzhusi Formation, and the Canglangpu Formation (Figure 1 d).^29,30^ Among these, the lithology of the Doushantuo Formation (635–550 Ma) is mainly composed of siltstone and shale. The Dengying Formation (550–539 Ma) is in conformable contact with the underlying Doushantuo Formation. On the Middle-Yangtze Block, it is mainly composed of carbonate rocks; clastic interlayers increase toward the paleocontinent, while it laterally changes into contemporaneous heterofacies siliceous strata of the Liuchapo Formation in the Nanhua Basin. The Dengying Formation is divided into four members from bottom to top, and the lithological characteristics of each member are different: the first member of the Dengying Formation has micritic dolomite deposits; the second member of the Dengying Formation is dominated by algal dolomite sediments and is relatively thick; the third member of the Dengying Formation is dominated by mixed tidal-flat deposits; and the fourth member of the Dengying Formation is mainly composed of algal dolomite, with local development of siliceous dolostone.^31^ The Dengying Formation and the overlying Maidiping Formation (539–521 Ma) are usually in unconformable contact; the laminar microcrystalline dolomite and algal-laminated siliceous dolostone in the central part of the Maidiping Formation within the rift trough share the same isochronous stratigraphic framework with the lower interval of the Dengying Fourth Member on the platform. The high-phosphorus members of the upper part of the Maidiping Formation in the rift trough and the upper member of the fourth member of Dengying main body in the platform were deposited in a synchronous and heterogeneous deep-water environment.^19^ The Qiongzhusi Formation (dating from 521 to 517 million years ago) has an unconformable contact with the Maidiping Formation. It is the most important source rock and cap rock in the Ediacaran–Cambrian petroleum system of the Sichuan Basin. The bottom of the Canglangpu Formation (dating from 517 to 511 million years ago) is characterized by the development of dolomite, while the upper part is dominated by clastic deposits.

## 3 Samples and Methods

### 3.1 Sample Characteristics

The chert samples used in this study were collected from the cores of Section B1 and Well A1 in the central part of the study area. The latitude and longitude coordinates of the B1 section are 106°22′46.542″E and 32°30′39.1968″N. Silica rock occurs in the stratum of the fourth member of the Sinian Dengying Formation and is associated with medium-thick algal dolomite.

Siliceous sediment samples for this study were collected from the the fourth member of the Sinian Dengying Formation strata in the B1 outcrop section and A1 well core in the central part of the study area. Meanwhile, clastic zircon U–Pb dating samples were collected from the DL outcrop section, also in the central study area. The main lithologies in the fourth member of the Dengying Formation (Deng 4) in Well A1 and Section B1 are algal dolomite, muddy siltstone dolomite, and siliceous dolostone. The siliceous dolostone deposits are observed in the middle-lower and top parts of Submember Deng 4I, and in the middle and top parts of Submember Deng 4II in Section B1. Additionally, algal boundstone dolomite with well-developed dissolution vugs is present at the top of Submember Deng 4II in Well A1, indicating karstification at the top surface. This suggests that the siliceous dolostone at the top of Section B1 may have been filled by later silica-rich fluids. In the field outcrop of Section B1, siliceous dolostone is associated with medium-thick bedded algal dolomite. The weathered surfaces of the rocks appear yellowish-brown to brown, while fresh surfaces are dark gray to black. The siliceous dolostone occurs in layered, nodular, and banded forms with individual bed thicknesses ranging from several centimeters to tens of meters (Figure 2a–c). Under the microscope, the chert exhibits a cryptocrystalline to fine-grained texture and is predominantly composed of cryptocrystalline and microcrystalline quartz (Figure 2d–g), and the particle sizes are 0.01–1 mm. Silica precipitated at different filling stages exhibits distinct macroscopic and microscopic occurrences and distribution patterns. Silica formed during the penecontemporaneous stage, which fills the margins of intergranular dissolution pores in algal dolomite, generally occurs as cryptocrystalline silica, followed by granular quartz as the subsequent fill phase (Figure 2 d–g).

**Figure 2:**
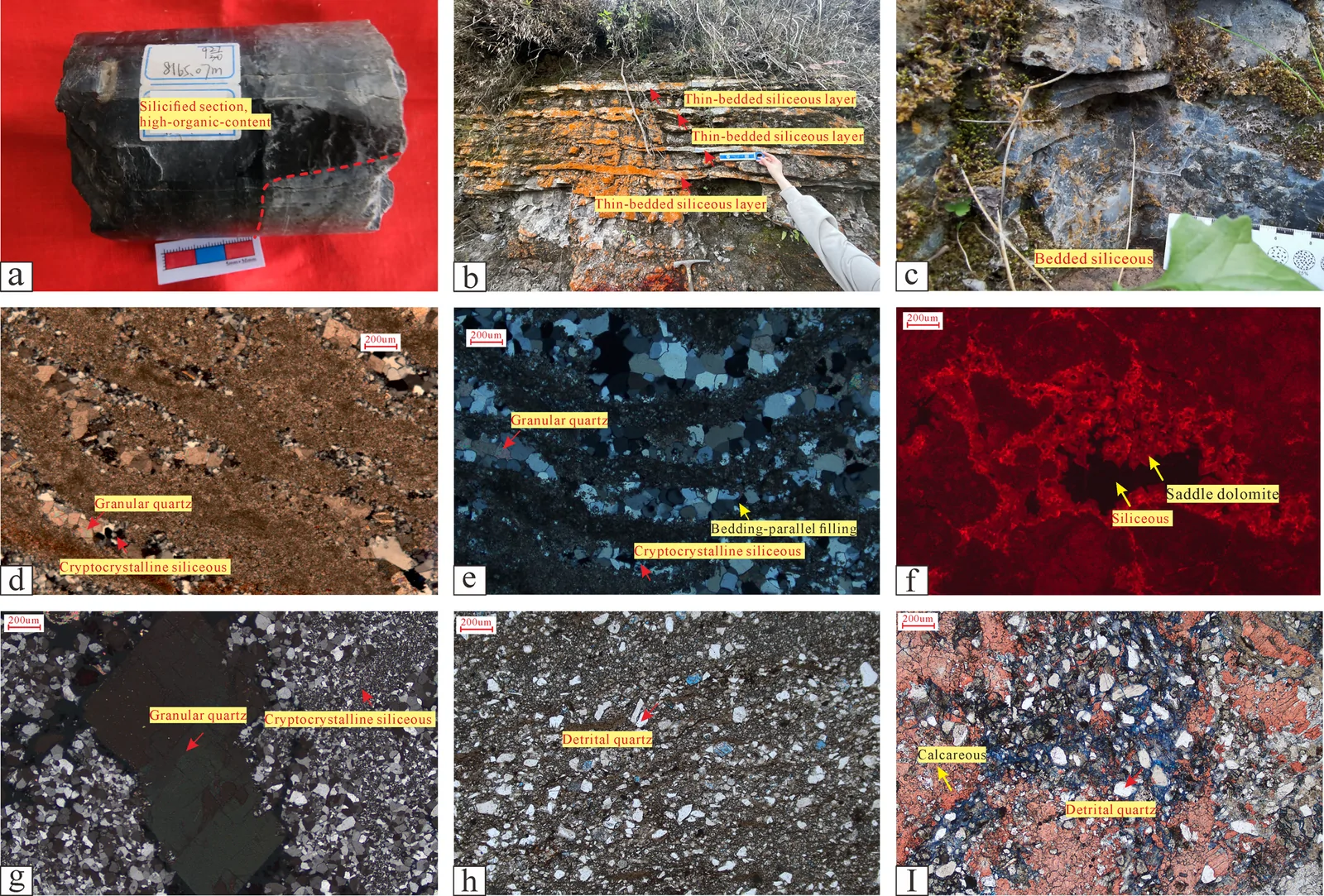
Characteristics of siliceous occurrences in the northwestern Yangtze Block (a) A1 well, 8165.07 m, chert, with granular quartz filling inside the dissolution pores; (b) B1 profile, PM B1-16, 29 layers, with thin-bedded siliceous material interbedded between algal dolomite layers; (c) B1 profile, PM B1-17, 33 layers, bedded chert; (d) A1 well, 8165.4 m, siliceous dolostone, bedding-parallel filling of siliceous material, cryptocrystalline siliceous filling, with granular quartz filling along the margins of dissolution pores, orthogonal polarization; (e) A1 well, 8164.91 m, siliceous dolostone, bedding-parallel filling of siliceous material, orthogonal polarization; (f) B1 profile, PM B1-13, 79.32 m, siliceous dolostone, with siliceous filling and saddle dolomite interior, cathodoluminescence; (g) B1 profile, PM B1-8, 142.37 m, chert, with intergranular dissolved pores, which are filled with coarse quartz particles, and the bedrock is cryptocrystalline siliceous with orthogonal polarization; (h) B1 profile, 53.82 m, sandy dolomite, with detrital quartz and siliceous debris, single-polarized light; (i) B1 profile, 249.97 m, sandy calcareous dolomite, with detrital quartz and siliceous detritus, single-polarized light.

In this study, chert and siliceous dolostone in the samples were collectively termed as siliceous sediments. A total of 21 fresh lithogeochemical samples from Member 4 of the Dengying Formation in the northwestern Yangtze Block were collected, including section B1: 5 chert, 4 siliceous dolostones, 4 algal dolomites, and 3 micritic to silty dolomites. Among these, 13 samples underwent whole-rock compositional analysis; all samples were tested for major, trace, and rare earth elements (REEs), and 5 siliceous sediment samples were analyzed for silicon–oxygen isotopes.

Well A1: 3 chert, 1 siliceous dolostone, and 1 algal dolomite, with all samples subjected to silicon–oxygen isotope studies. Care was taken to exclude and avoid contamination from adjacent strata and extraneous materials. All samples were unweathered, and their specific sampling locations are shown in Figure 3. For detrital zircon U–Pb dating of the Canglangpu Formation, one pebbly siltstone sample was collected from the DL section. The detrital zircon U–Pb geochronological data for this sample from the DL section are derived from a contemporaneous regional provenance study conducted by our research group.^32^ This paper mainly relies on these age constraints to discuss the sedimentary and tectonic settings of siliceous sediments.

**Figure 3:**
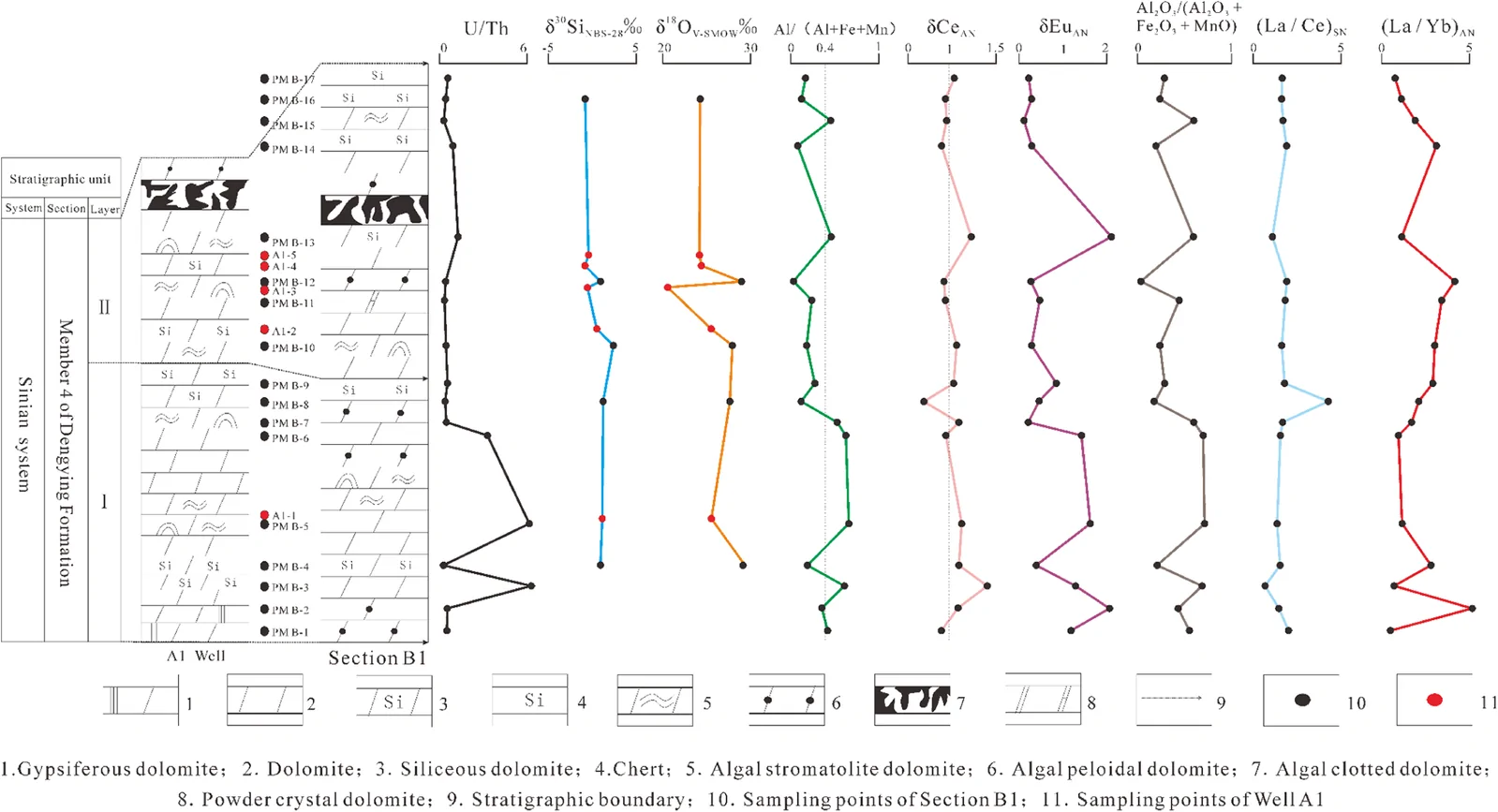
Sampling points and geochemical characteristics of siliceous sediments in the northwestern Yangtze Block.

### 3.2 Analytical Methods

In this study, the main tests included whole-rock X-ray diffraction (XRD), major and trace element tests, Si and O isotope tests, and detrital zircon U–Pb dating analysis of the samples. According to the national standard SY/T5163-2018 “X-ray diffraction analysis of clay minerals and common nonclay minerals in sedimentary rocks,” the samples were subjected to XRD analysis using an MS1-044-1 X-ray diffractometer, and the analysis error was less than 5%. According to GB/T14506.028-2010 “Methods of Chemical Analysis of Silicate Rocks-Part 28: Determination of the Quantities of 16 Primary and Secondary Components,” the samples from the study area were analyzed using an MS2-006-1 X-ray fluorescence spectrometer (AXios-mAX). For elemental determination, the analytical accuracy was less than 2–5%. According to GB/T14506.30-2010 “Methods for chemical analysis of silicate rocks-Part 30: Determination of 44 elements,” the rare earth elements and trace elements of the samples from the study area were analyzed using an MS2-068 inductively coupled plasma mass spectrometer (iCAP RQ). For element determination, the accuracy of the elements with contents greater than 10 × 10^–6^ was 5%, whereas the accuracy of the elements with contents less than 10 × 10^–6^ was 10%. According to DZ/T0184.22-1997 “Determination of Silicon Isotopic Composition” and DZ/T0184.13-1997 “Determination of Bromine Pentafluoride in Silicate and Oxide Minerals,” an MS605 gas isotope ratio mass spectrometer 253 Plus was used to determine the silicon and oxygen isotopes of the samples from the study area. The specific procedures of the silicon isotope test were as follows: first, the sample was crushed to a mesh size of 200–300, then soaked in dilute hydrochloric acid, rinsed repeatedly with distilled water, dried in air, purified, and then oxidized. SiO_2_ was treated with purified BrF_5_ and was oxidized to SiF_4_.^33^ The quartz sand sample standard NBS-28 of the American National Bureau of Standards was used for the reference samples, with an analytical accuracy of ±0.1‰. Oxygen isotopes were also prepared by the bromine pentafluoride method.^33^ The reaction principle was as follows: silicate or oxide reacted with the strong oxidant BrF_5_ at high temperature to form O_2_; O_2_ then reacted with a carbon rod and was converted to CO_2_. The reference sample was SMOW, and the analytical accuracy was ±0.2‰. The above tests were all performed at Sichuan Keyuan Engineering Technology Testing Center Co., Ltd.

Zircon grains were separated from the samples by using conventional heavy mineral separation techniques. Pure zirconium grains were obtained through magnetic separation and heavy liquid separation. All experiments and subsequent data processing were conducted at Nanjing Shunke Testing Technology Co., Ltd., which is equipped with a RESOlution SE laser ablation system and an iCAP RQ ICP-MS instrument. In the analysis, the international reference zircon 91500 (age of 1065 Ma) was employed as the external reference for correcting U–Pb isotope fractionation effects and mass discrimination. Additionally, a secondary zirconium reference sample served as a monitoring blind sample to assess the stability of the testing process. During the analysis, a set of reference samples was inserted after every five sample points to ensure the accuracy of the analytical data; the accuracy of the ^206^Pb/^238^U age test was better than 2%,^34^ while the accuracy of the ^207^Pb/^206^Pb age test was better than 5%. Data reduction was conducted using software ICPMSDataCal.^35^

In this study, the anomalous expressions of cerium and europium are as follows: δCe = 2Ce_AN_/(La_AN_ + Pr_N_) and δEu = 2Eu_AN_/(Sm_AN_ + Gd_AN_). The rare earth element partition model used the average post-Archean shale in Australia (PAAS) for standardization. δCe_CN_, δEu_CN_, (La/Yb)_CN_, and (La/Ce)_CN_; δCe_SN_, δEu_SN_, (La/Yb)_SN_, and (La/Ce)_SN_; and δCe_AN_, δEu_AN_, (La/Yb)_AN_, and (La/Ce)_AN_ represent the Ce outlier values, Eu outlier values, and the La/Yb and La/Ce ratios for rare earth elements normalized to North American shale,^36^ chondrite, and post-Archean shale in Australia, respectively.^37^

## 4 Results

### 4.1 Whole-Rock Characteristics

The results of the whole-rock composition analysis of the geochemical samples from the B1 profile in the northern Sichuan area are listed in Table 1.^38^ In this study, based on the differences in the rock composition and silica content, the lithology of the fourth member of the Dengying Formation in the study area was divided into chert, siliceous dolostone, and algal dolomite. The SiO_2_ content of chert was 82.1–97.2%, SiO_2_ content of siliceous dolostone was 54.2–63%, and the SiO_2_ content of algal dolomite and micritic to silty dolomite was less than 50%. The siliceous dolostone and chert with a siliceous mineral content greater than 50% are called siliceous sediments, and they are the main research objects of this paper.

**Table 1:** Analysis Results of the Mineral Composition of Siliceous Sediments and Dolomite in the Northwestern Yangtze Block

Number	Lithology	Quartz	K-feldspar	Plagioclase	Calcite	Dolomite	Fluorite	Siderite	Iron pyrite	Anatase	Anhydrite	Clay mineral
PM B1-1	Siliceous dolostone	54.2	0.5	0.0	30.5	12.7	0.0	0.0	0.0	0.0	2.1	0.0
PM B1-4	Siliceous dolostone	62.4	0.0	0.0	0.5	37.1	0.0	0.0	0.0	0.0	0.0	0.0
PM B1-7	Micritic to silty dolomite	3.8	0.0	0.0	0.0	96.2	0.0	0.0	0.0	0.0	0.0	0.0
PM B1-8	chert	99.2	0.0	0.0	0.0	0.8	0.0	0.0	0.0	0.0	0.0	0.0
PM B1-9	chert	94.7	0.0	0.0	0.4	4.2	0.7	0.0	0.0	0.0	0.0	0.0
PM B1-10	Siliceous dolostone	59.0	0.0	0.0	0.0	41.0	0.0	0.0	0.0	0.0	0.0	0.0
PM B1-11	Algal dolomite	28.4	0.0	0.0	0.0	71.6	0.0	0.0	0.0	0.0	0.0	0.0
PM B1-12	Algal dolomite	9.0	0.0	0.0	0.0	91.0	0.0	0.0	0.0	0.0	0.0	0.0
PM B1-13	Siliceous dolostone	63.0	10.0	0.0	0.0	0.0	0.0	2.0	3.0	0.0	0.0	22.0
PM B1-14	Chert	82.1	0.0	0.0	0.4	17.5	0.0	0.0	0.0	0.0	0.0	0.0
PM B1-15	Algal dolomite	45.2	0.0	0.0	0.0	54.8	0.0	0.0	0.0	0.0	0.0	0.0
PM B1-16	Chert	97.1	0.0	0.0	0.0	2.9	0.0	0.0	0.0	0.0	0.0	0.0
PM B1-17	Chert	97.2	0.0	0.0	1.5	1.3	0.0	0.0	0.0	0.0	0.0	0.0

The chert contains some dolomite and calcite and almost no metallic or clay minerals. The dolomite content in the siliceous dolostone increases, and the contents of metal minerals and clay minerals are generally <3%. Only some clay minerals and anhydrite can be found in PM B1-1 and PM B1-13, with contents of 2.1% and 22%, respectively; some siderite and pyrite are found in PM B1-13, with a content of 5%.

### 4.2 Characteristics of Major Elements

The analysis results of the five chert samples showed that the SiO_2_ content of the fourth member of the Dengying Formation in the northern Sichuan area was 87.7–98.2%; the CaO and MgO contents were relatively high, at 0.2–2.5% and 0.3–3.7%, respectively; the contents of Al_2_O_3_ and Fe_2_O_3_ were generally low, at 0.1–0.6% and 0.2–0.9%, respectively; and the other contents were very low and less than 1% (Table 2).^38^ Analysis of the four pieces of siliceous dolostone from the fourth member of the Dengying Formation in the northern Sichuan area showed that the SiO_2_ content of the deposited siliceous dolostone was 61.6–68.1%; compared with those of the chert, the CaO and MgO contents of the siliceous dolostone significantly increased and were 9.9–16.7% and 2.6–8.7%, respectively. Compared with chert, siliceous dolostone had significantly higher contents of Al_2_O_3_ and Fe_2_O_3_, at 0.1–1.2% and 0.1–1.1%, respectively; the other contents were very low and less than 1%.

**Table 2:** Main-Element Content (%) Data of Siliceous Sediments in the Northwestern Yangtze Block

Lithology	Number of chert					Number of siliceous dolostone			
Element	PM B1-8	PM B1-9	PM B1-14	PM B1-16	PM B1-17	PM B1-1	PM B1-4	PM B1-10	PM B1-13
SiO_2_ (%)	98.2	92.9	87.7	97.4	95.7	62.1	68.1	61.6	65.1
Al_2_O_3_ (%)	0.1	0.4	0.3	0.4	0.6	1.2	0.1	0.1	0.2
MgO (%)	0.2	0.7	2.5	0.3	0.2	2.6	7.0	8.7	7.0
Na_2_O (%)	0.0	0.1	0.0	0.1	0.1	0.0	0.0	0.0	0.4
K_2_O (%)	0.0	0.1	0.0	0.1	0.1	0.6	0.0	0.0	0.2
P_2_O_5_ (%)	0.0	0.0	0.0	0.0	0.0	0.1	0.0	0.0	0.0
TiO_2_ (%)	0.0	0.0	0.0	0.0	0.0	0.0	0.0	0.0	0.1
CaO (%)	0.3	1.8	3.7	0.5	0.7	16.7	9.9	12.0	14.1
TFe_2_O_3_ (%)	0.2	0.6	0.7	0.7	0.9	1.1	0.2	0.3	0.1
MnO (%)	0.0	0.1	0.1	0.1	0.1	0.0	0.0	0.0	0.0
Lol (%)	0.8	2.3	5.7	1.0	1.3	15.6	15.0	18.6	12.1
Tatol (%)	99.8	99.0	100.8	100.5	99.6	100.0	100.3	101.3	99.4
Si/Al	1042.9	194.6	300.9	220.2	138.3	46.5	652.0	434.0	260.6
Fe/Ti	27.5	56.2	57.5	58.0	51.7	36.4	22.4	29.3	2.1
CIA	25.0	21.6	6.9	65.0	71.8	6.8	0.9	1.1	1.5
Al/(Al + Fe + Mn)	0.2	0.3	0.2	0.3	0.3	0.4	0.2	0.3	0.6
Si/(Si + Al + Fe)	1.0	1.0	1.0	1.0	1.0	1.0	1.0	1.0	1.0
(Fe + Mn)/Ti	30.5	61.4	62.5	63.2	55.8	38.0	25.5	32.8	2.3
SiO_2_/MgO	609.9	128.0	35.3	325.9	566.3	24.3	9.7	7.1	9.3
Al_2_O_3_/TiO_2_	34.7	32.4	17.1	27.9	30.5	34.7	92.0	12.5	3.4
Fe_2_O_3_/TiO_2_	23.6	48.2	49.3	49.7	44.3	31.2	192.0	25.1	1.8
Al_2_O_3_/(Al_2_O_3_ + Fe_2_O_3_)	0.3	0.4	0.3	0.4	0.4	0.5	0.3	0.3	0.7
Al_2_O_3_/(Al_2_O_3_ + Fe_2_O_3_ + MnO)	0.2	0.4	0.2	0.3	0.4	0.5	0.3	0.3	0.6

### 4.3 Trace Element Characteristics

According to the trace element analysis results and parameters of the siliceous sediments (Table 3),^38^ some trace elements, such as Nb, Th, Rb, U, Ni, Cr, Zr, and V, are effective indicators for determining the origin of siliceous sediments.^9,37^ The siliceous sediments from the northern Sichuan area were analyzed for 26 trace elements, including Cr, Ni, Rb, and Ba. The mean Cr content was 32.3 μg/g (15.5–48.2 μg/g), and the mean Zr content was 14.0 μg/g (1.0–100.0 μg/g). The mean Ba, Nb, Rb, and Th contents were 167.4 μg/g (17.0–922.0 μg/g), 0.6 μg/g (0.2–1.1 μg/g), 2.5 μg/g (0.8–9.4 μg/g), and 0.9 μg/g (0.3–1.9 μg/g), respectively. The above trace element contents were low and were in agreement with the characteristics of a hydrothermal origin.

**Table 3:** Analysis Results and Parameter Values of Rare Earth Elements and Trace Elements in Siliceous Sediments in the Northwestern Yangtze Block^a^

Lithology	Number of chert					Number of siliceous dolostone						
Element	PM B1-8	PM B1-9	PM B1-14	PM B1-16	PM B1-17	PM B1-1	PM B1-4	PM B1-10	PM B1-13	A	B1	B2
Sc	0.1	0.1	0.1	0.2	0.3	1.7	0.2	0.1	9.8	16.0	0.0	0.2
La	2.1	2.5	2.8	0.9	1.1	4.6	3.1	1.7	1.3	28.7	0.1	0.1
Ce	1.2	3.2	3.2	1.4	1.7	6.0	4.6	2.4	2.7	6.2	0.4	0.6
Pr	0.4	0.4	0.4	0.2	0.2	1.0	0.5	0.3	0.3	7.2	0.1	0.1
Nd	1.4	1.4	1.3	0.8	0.8	4.1	1.9	1.0	0.8	28.7	0.0	0.1
Sm	0.3	0.2	0.2	0.1	0.2	0.8	0.3	0.2	0.4	6.9	0.0	0.1
Eu	0.1	0.1	0.0	0.0	0.0	0.2	0.1	0.0	0.3	1.1	0.1	0.1
Gd	0.8	0.8	0.8	1.0	1.5	1.6	0.8	0.7	0.8	5.9	0.2	0.2
Tb	0.0	0.0	0.0	0.0	0.0	0.2	0.0	0.0	0.1	0.9	0.0	0.1
Dy	0.2	0.1	0.1	0.1	0.1	1.1	0.2	0.1	1.3	4.0	0.0	0.2
Y	1.3	1.2	1.1	1.2	1.5	8.4	1.4	0.9	4.6	27.7	0.1	0.1
Ho	0.0	0.0	0.0	0.0	0.0	0.3	0.0	0.0	0.0	1.0	0.0	0.1
Er	0.1	0.1	0.1	0.1	0.1	0.7	0.1	0.1	0.1	2.9	0.0	0.1
Tm	0.0	0.0	0.0	0.0	0.0	0.1	0.0	0.0	0.0	0.3	0.0	0.1
Yb	0.1	0.1	0.1	0.1	0.1	0.7	0.1	0.1	0.1	2.6	0.0	0.1
Lu	0.0	0.0	0.0	0.0	0.0	0.1	0.0	0.0	0.0	0.8	0.0	0.0
Th	0.3	1.6	1.9	0.4	0.4	0.8	1.3	1.0	0.6	7.6	0.1	0.1
Li	0.8	2.3	1.3	2.6	2.0	4.9	2.2	1.1	1.6	20.0	0.1	0.1
Be	0.0	0.1	0.0	0.0	0.1	0.2	0.1	0.0	0.1	2.8	0.0	0.0
V	1.3	3.0	2.6	3.9	4.1	6.8	9.2	4.8	3.2	143.0	0.0	0.0
Cr	15.5	48.2	37.7	39.3	43.0	35.4	21.2	22.5	27.8	127.0	0.3	0.2
Co	0.4	0.9	0.8	18.6	0.9	1.1	0.3	0.4	1.9	24.7	0.2	0.0
Ni	2.7	3.6	4.2	111.3	6.5	676.7	2.8	2.6	21.4	81.3	0.3	2.2
Cu	5.3	2.6	2.7	4.1	3.0	11.3	1.6	1.6	8.7	56.0	0.1	0.1
Zn	9.3	12.9	12.1	13.6	19.7	13.2	9.5	10.1	14.3	76.3	0.2	0.2
Ga	0.4	1.4	0.6	0.7	0.9	1.7	0.6	0.4	1.8	15.0	0.1	0.1
Rb	0.8	1.8	1.5	1.8	2.5	9.4	0.6	1.0	2.9	108.0	0.0	0.0
Sr	5.4	20.8	13.3	35.1	40.4	30.1	19.4	20.7	20.9	382.0	0.1	0.1
Zr	1.0	3.2	1.5	3.2	4.2	10.6	1.6	1.0	100.0	148.0	0.0	0.2
Nb	0.2	0.4	1.0	0.4	0.5	1.1	0.1	0.2	1.0	18.3	0.0	0.0
Mo	0.0	0.0	0.2	0.3	0.4	0.1	0.0	0.0	0.2	1.5	0.1	0.1
Cd	0.1	0.0	0.1	0.1	0.3	0.1	0.0	0.2	0.0	0.2	0.6	0.4
Ba	18.6	921.9	42.3	138.1	189.2	96.2	19.5	17.0	63.8	463.0	0.6	0.1
Hf	0.0	0.1	0.1	0.1	0.1	0.4	0.1	0.0	2.1	4.5	0.0	0.1
Pb	13.5	3.1	1.9	1.0	1.1	1.8	1.7	0.7	4.4	14.0	0.3	0.2
U	0.4	2.1	2.0	2.7	1.2	0.4	0.6	0.8	1.0	2.1	0.8	0.3
∑REE	8.0	10.2	10.2	6.0	7.3	30.0	13.2	7.3	12.6	—	—	—
LREE/HREE	0.5	0.7	0.6	0.3	0.3	0.3	0.7	0.5	0.6	—	—	—
∑REE/Fe	4.4	2.0	1.5	1.2	1.3	4.4	6.8	3.2	15.8	—	—	—
δCe_CN_	0.3	0.7	0.6	0.7	0.8	0.6	0.8	0.7	1.0	—	—	—
δEu_CN_	0.5	1.1	0.5	0.3	0.3	1.0	0.5	0.3	2.6	—	—	—
δCe_SN_	0.3	0.7	0.6	0.7	0.8	0.6	0.8	0.7	1.0	—	—	—
δEu_SN_	0.3	0.7	0.3	0.2	0.2	0.6	0.3	0.2	1.6	—	—	—
δCe_AN_	0.3	0.7	0.7	0.7	0.8	0.6	0.8	0.8	1.0	—	—	—
δEu_AN_	0.5	0.9	0.4	0.3	0.2	0.9	0.4	0.3	2.2	—	—	—
La/Ce	1.7	0.8	0.9	0.6	0.7	0.8	0.7	0.7	0.5	—	—	—
(La/Yb)_CN_	2.8	3.5	4.2	1.4	1.1	0.7	3.6	3.7	2.4	—	—	—
(La/Ce)_CN_	3.8	1.8	2.0	1.5	1.5	1.8	1.5	1.6	1.1	—	—	—
(La/Yb)_SN_	19.8	24.5	29.3	9.5	7.8	4.6	25.1	25.4	16.9	—	—	—
(La/Ce)_SN_	4.4	2.0	2.3	1.7	1.7	2.0	1.7	1.9	1.3	—	—	—
(La/Yb)_AN_	2.2	2.7	3.2	1.0	0.9	0.5	2.7	2.8	1.8	—	—	—
(La/Ce)_AN_	3.5	1.6	1.9	1.3	1.4	1.6	1.4	1.5	1.0	—	—	—
U/Th	1.6	1.4	1.1	6.4	2.7	0.5	0.5	0.8	1.5	—	—	—
Sr/Ba	0.3	0.0	0.3	0.3	0.2	0.3	1.0	1.2	0.3	—	—	—
Ba/Eu	349.9	8341.0	972.9	3938.7	4443.3	415.3	379.2	590.5	209.9	—	—	—
Ni/Co	6.5	3.9	5.1	6.0	7.2	602.3	8.4	6.8	11.5	—	—	—
V/Y	1.0	2.5	2.3	3.3	2.8	0.8	6.4	5.5	0.7	—	—	—
Y/Ho	1.4	1.6	2.2	1.8	1.9	1.2	1.7	2.0	8.4	—	—	—

a Note: A— abundance values of crustal elements; B1— enrichment coefficient of chert elements; B2—element enrichment coefficient of siliceous dolostone; δCe_CN_, δEu_CN_, (La/Yb)_CN_, and (La/Ce)_CN_; δCe_SN_, δEu_SN_, (La/Yb)_SN_, and (La/Ce)_SN_; and δCe_AN_, δEu_AN_, (La/Yb)_AN_, and (La/Ce)_AN_ are the standardization of rare earth elements in North American shale,^36^ chondrite meteorite standardization, and the standardization of La/Yb ratio, Eu anomaly, and Ce anomaly in post Archean shale in Australia.

### 4.4 Characteristics of the Rare Earth Elements

According to the rare earth element analysis results and parameters of the siliceous sediments (Table 3),^38^ the Y/Ho values of the samples ranged from 1.4 to 8.4; these results indicated that the two elements, Y and Ho, did not significantly differ. The total amount of rare earth elements (∑REE) was low, with a range of 6.0–30.0 μg/g and an average value of 11.6 μg/g; these results indicated that the siliceous sediments in the study area were deposited in a relatively deep-water environment. The ratio of the light and heavy REE contents (∑LREE/HREE) was 0.5, with a range of 0.3–0.7 The average Ce_AN_ anomaly was 0.7, with a range of 0.3–1.0; these results indicated negative Ce anomaly as a whole. The average Eu_AN_ anomaly was 0.7, with a range of 0.2–2.2; this showed a partially positive Eu anomaly. The (La/Yb)_AN_ ratio was 0.9–3.2, with an average of 2.0. The (La/Ce)_AN_ ratio was 1.0–3.5, with an average of 1.7.

### 4.5 Silicon and Oxygen Isotope Characteristics

The study of silica and oxygen isotopes is highly important for determining the source and cause of silica. The δ^30^Si_NBS‑28_‰ values of the chert were 0.6–0.9‰, and the δ^18^O_V‑SMOW_‰ values of the chert were 24.2–28.0‰. The δ^30^Si_NBS‑28_‰ values of the siliceous dolostone were 0.7–3.3‰, and the δ^18^O_V‑SMOW_‰ values were 25.8–29.0‰. The δ^30^Si_NBS‑28_‰ values of the algal dolomite were −0.3–0.6‰, and the δ^18^O_V‑SMOW_‰ values were 20.4–29.3‰ (Table 4).^38^

**Table 4:** Results of Silicon–Oxygen Isotope Analysis of Siliceous Sediments in the Northwestern Yangtze Block

Sample number	Well/section	Lithology	δ^18^O_V‑SMOW_‰	δ^30^Si_NBS‑28_‰
PM B1-4	B1 section	Siliceous dolostone	29.0	0.7
PM B1-10	B 1 section	Siliceous dolostone	28.0	3.3
A1-1	A1 well	Siliceous dolostone	25.8	0.7
PM B1-8	B 1 section	Chert	28.0	0.9
PM B1-16	B 1 section	Chert	24.4	–0.6
A1-2	A1 well	Chert	25.5	0.1
A1-4	A1 well	Chert	24.8	–0.4
A1-5	A1 well	Chert	24.2	–0.3
PM B1-12	B 1 section	Algal dolomite	29.3	0.6
A1-3	A1 well	Algal dolomite	20.4	–0.3

## 5 Discussion

### 5.1 Tectonic Setting During the ECT Period

Zircons formed in distinct modern tectono-magmatic settings can be discriminated by analyzing the internal trace element ratios (e.g., U, Th, Nb, Yb) within zircon grains.^39,40^ Zircons predating the Neoproterozoic from the study area predominantly yield continental arc signatures, together with features indicative of arc-related orogenic compression and contributions from depleted mantle sources (Figure 4a–c).^39–41^ These geochemical signals are closely associated with subduction and extensional events along the northwestern margin of the Yangtze Block during the breakup of the Rodinia supercontinent and the assembly of Gondwana. It is further confirmed that zircons from the study area crystallized under arc-related tectonic conditions, satisfying the prerequisite for crustal thickness estimation.

**Figure 4:**
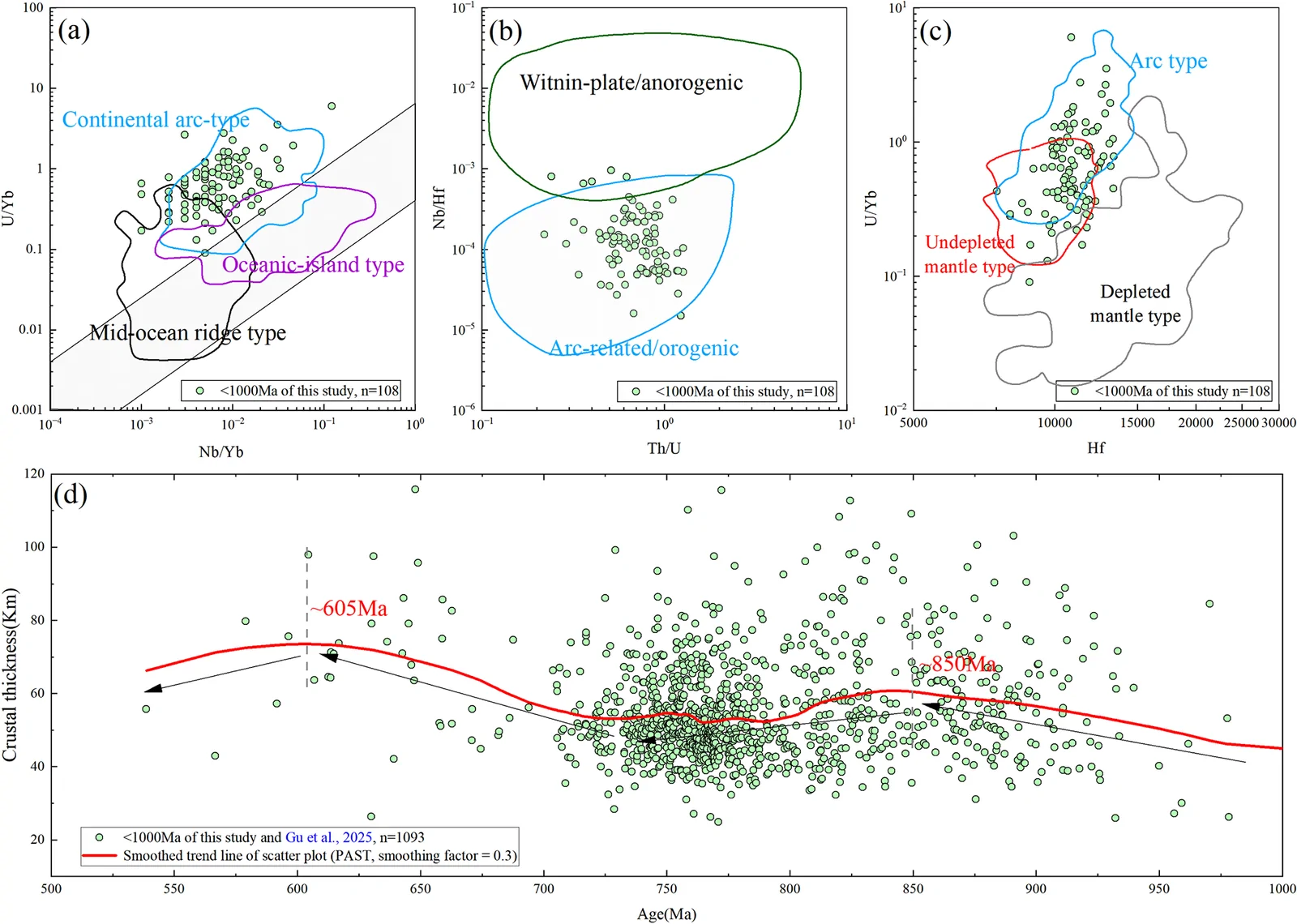
Trace element discrimination diagrams of Neoproterozoic zircon grains and crustal thickness evolution on the northwestern margin of the Yangtze Block. (a) Nb/Yb vs U/Yb diagram for zircons younger than 1.0 Ga; (b) Th/U vs Nb/Hf diagram for zircons younger than 1.0 Ga; (c) Hf vs U/Yb diagram for zircons younger than 1.0 Ga; (d) Crustal-thickness evolution calculated from zircon Eu/Eu* ratios for zircons younger than 1.0 Ga.

Detrital zircon U–Pb geochronological data of sample DL38 are derived from a contemporaneous regional provenance study conducted by our research group.^32^ This paper mainly relies on these age constraints to discuss the tectonic setting during deposition of siliceous sediments. On this basis, we calculate the evolutionary trend of crustal thickness on the northwestern Yangtze Block using detrital zircon Eu/Eu* ratios following the method of Gu et al. (2025).^41^ The results reveal two prominent crustal thinning episodes after the Neoproterozoic along the northwestern Yangtze Block, occurring at ∼850 Ma and ∼605 Ma, respectively. The latter thinning event coincides with the evolutionary interval of the ECT period, corroborating the initiation of arc-related extensional rifting in the study area after ∼605 Ma.

Furthermore, the mean value of Cr in the study area was 32.3 μg/g (15.5–48.2 μg/g), the mean Zr value was 14.0 μg/g (1.0–100.0 μg/g), and the contents of Zr and Cr were low; these results indicated that the depositional environment of the siliceous sediments in the study area was a continental margin.^42^ The V/Y ratio of chert in the study area ranges from 0.7 to 6.4, with an average of 2.8, which is close to that of continental margin chert. In the 100Fe_2_O_3_/SiO_2_–100Al_2_O_3_/SiO_2_ diagram,^42^ some samples also fall within the continental margin field (Figure 5). Combined with the island-arc signature of zircon grains, these results indicate that the study area was a continental marginal basin adjacent to an ancient volcanic arc.

**Figure 5:**
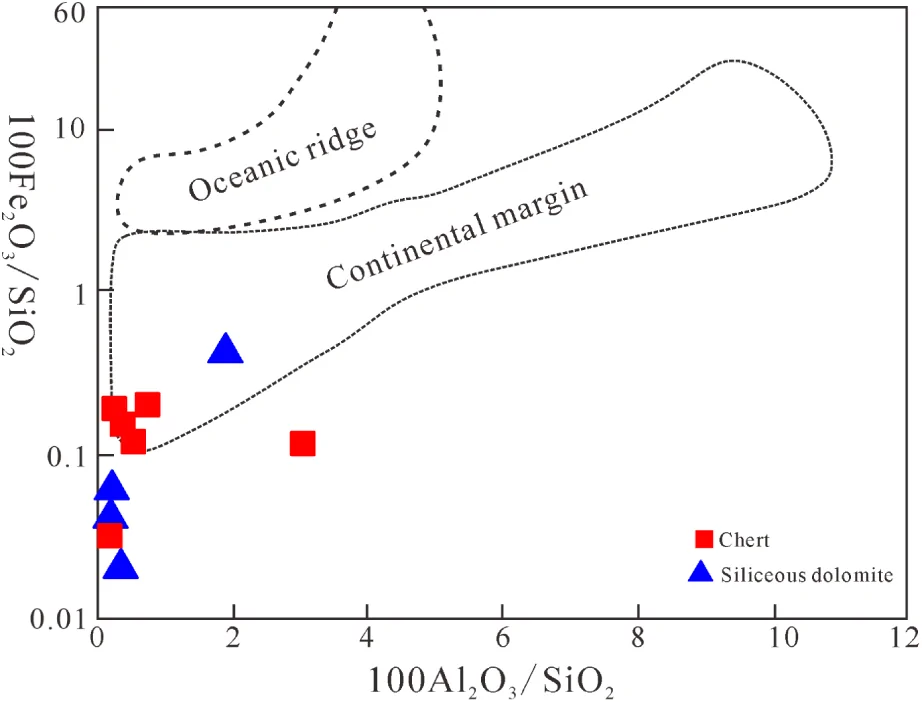
Schematic diagram of sedimentary environment discrimination of siliceous sediments in northern Sichuan.

### 5.2 Genetic Analysis and Sedimentary Environment of Siliceous Sediments

#### 5.2.1 Constraints of Silicon and Oxygen Isotopes on Biogenic Origin

The loss on ignition (LOI) of the siliceous sediments in the study area is generally low. All siliceous rock samples exhibit consistent and well-parallel rare-earth-element (REE) distribution patterns, with systematic variations in Ce anomalies and a narrow range of LREE/HREE ratios ranging from 0.3 to 0.7. These features indicate that primary geochemical signatures are well preserved and barely modified by late weathering and diagenesis (Table 3). Furthermore, the low chemical index of alteration (CIA) values (Table 2) further confirm that the analyzed samples are fresh enough to reliably reconstruct the paleoenvironmental conditions and material sources during sedimentation.

The δ^30^Si value of siliceous sediments can reflect the source of silica in different sedimentary environments. The concentrations and silicon isotopes of dissolved silicon in seawater at different depths were different. Dissolved silicon in the shallow sea area close to land likely had a relatively high δ^30^Si value in the abyssal area farther from the land because due to the greater distance from the river water supply, the contributions from the seafloor hydrothermal activity, the seafloor rock alteration, and the silica precipitation from the biological remains were greater, and the δ^30^Si value was lower.^43^ Studies have shown that the δ^30^Si values of silica rock deposited in shallow marine environments were −0.4–3.4‰, with an average value of 1.3‰. The δ^30^Si values of the silica rock deposited in semi-deep-sea environments were 0.1–0.6‰, with an average value of 0.4‰. The δ^30^Si values of deep-water chert deposited in abyssal environments range from −0.6 to 0.8‰, with an average value of 0.2‰.^44,45^ The δ^30^Si_NBS‑28_‰ value of the chert in the study area ranged from −0.6 to 0.9‰, with an average value of −0.04‰. The δ^30^Si_NBS‑28_‰ value of the siliceous dolostone ranged from 0.7 to 3.3‰, with an average value of 1.6‰ (Table 4). The chert was closer to the source of silica in the deep-sea environment, and the siliceous dolostone was closer to the source of silica in the shallow-sea environment.

The δ^30^Si values of the Deng 4 Member chert in the study area were −0.6–0.9‰; these values were significantly different from those of metasomatic silicate and volcanic-eruption chemical sedimentary silicate and were close to the silicon isotopic composition range of biogenic chert. The δ^30^Si‰ value characteristics of the chert were similar to those of the Cambrian bottom chert, benthic radiolarian chert, and shallow-sea radiolarian chert in Xingdi, Xinjiang. The δ^30^Si values of the Deng 4 Member siliceous dolostone in the study area were −0.7–3.3‰; these values were closest to those expected for silica of metasomatic origin in silicified dolomite and were similar to those of chert or silicified dolomite and silicified stromatolite (Figure 6a).

**Figure 6:**
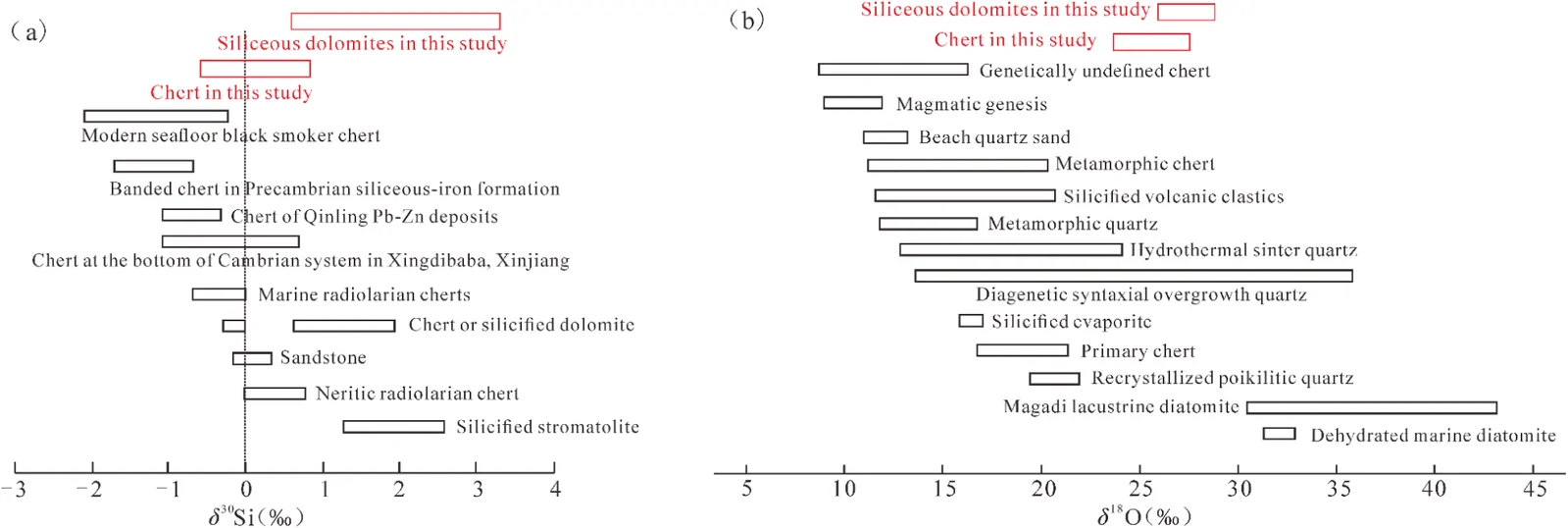
Silicon and oxygen isotope distributions of siliceous sediments of different origins. (a) Silicon isotope distribution. (b) Oxygen isotope distribution.

The δ^18^O‰ values of the Deng 4 Member chert and siliceous dolostone in the study area ranged from 24.2 to 29.0‰, and the δ^18^O‰ values of chert and siliceous dolostone were significantly different with those of replacement quartz, igneous quartz, metamorphic quartz and modern beach quartz. These values were mainly distributed in the range of δ^18^O‰ values for diagenetic secondary large quartz (Figure 6b) and were close to the values of primary chert and zeolite.^46^ In summary, the silica in cherts is derived from relatively deep-water environments and is closely related to biogenic processes. In contrast, silica within siliceous dolostone originates from relatively shallow marine settings and is mainly formed via metasomatism.

#### 5.2.2 Weak Hydrothermal Overprinting Signals Recorded by Trace Elements

Silicon and oxygen isotopic ratios can be used to distinguish the paleowater-depth environments of cherts and siliceous dolomites, yet the genetic mechanisms controlling the two lithologies differ significantly. Previous studies have shown that the values of Fe/Ti, (Fe + Mn)/Ti, Al/(Al + Fe + Mn), Fe/Ti–Al/(Al + Fe + Mn), Al_2_O_3_/TiO_2_, ∑REE, and δ^30^Si and δ^18^O can provide evidence for a siliceous origin. Fe/Ti > 20 and (Fe + Mn)/Ti > (20 ± 5) for typical modern marine hot-water sediments.^47^ Here, for the chert in the study area, Fe/Ti = 27.5–58.0, and the average value was 50.2; (Fe + Mn)/Ti = 30.5–63.2, and the average value was 54.7. For siliceous dolostone in the study area, Fe/Ti = 2.1–36.4, and the average value was 22.6; (Fe + Mn)/Ti = 2.3–38.0, and the average value was 24.6. These results indicated that the siliceous sediments experienced partial hydrothermal fluid overprinting during sedimentary and diagenetic processes, rather than being directly deposited by hydrothermal fluids.

The Al/(Al + Fe + Mn) ratio of pure hydrothermal sediments was approximately 0.01, and the Al/(Al + Fe + Mn) ratio of sediments affected by hydrothermal liquids was usually less than 0.4. An Al/(Al + Fe + Mn) ratio greater than 0.4 indicates a terrestrial source input, whereas this ratio was close to 0.6 for the chert formed by purely biological processes .^48^ The Al/(Al + Fe + Mn) values of chert in the study area ranged from 0.2 to 0.3, with an average value of 0.3; the Al/(Al + Fe + Mn) values of siliceous dolostone ranged from 0.2 to 0.6, with an average value of 0.4; these results further demonstrated that the study area’s siliceous sediments were dominated by biogenic seawater deposition, with only limited and localized hydrothermal fluid modification.

The Nb, Th, and Rb contents of the siliceous sediments in the study area were low, with average values of less than 3.5 μg/g. Moreover, no linear relationship was observed between the Al_2_O_3_ content and the Ni and Cr contents in the siliceous sediments (Figure 7a–c). These findings further indicated that the terrigenous clastics contributed little to the silica in the siliceous sediments. On the rare earth partition model map (Figure 7d–f), the chondrite-normalized curve showed negative anomalies for Ce and Eu, whereas the normalized curve for North American shale and post-Archean shale in Australia showed a weak positive anomaly of Eu and a negative anomaly of Ce; these results indicated that the siliceous sedimentary system underwent sporadic hydrothermal fluid perturbation and late diagenetic alteration, without pervasive basin-scale hydrothermal sedimentation.

**Figure 7:**
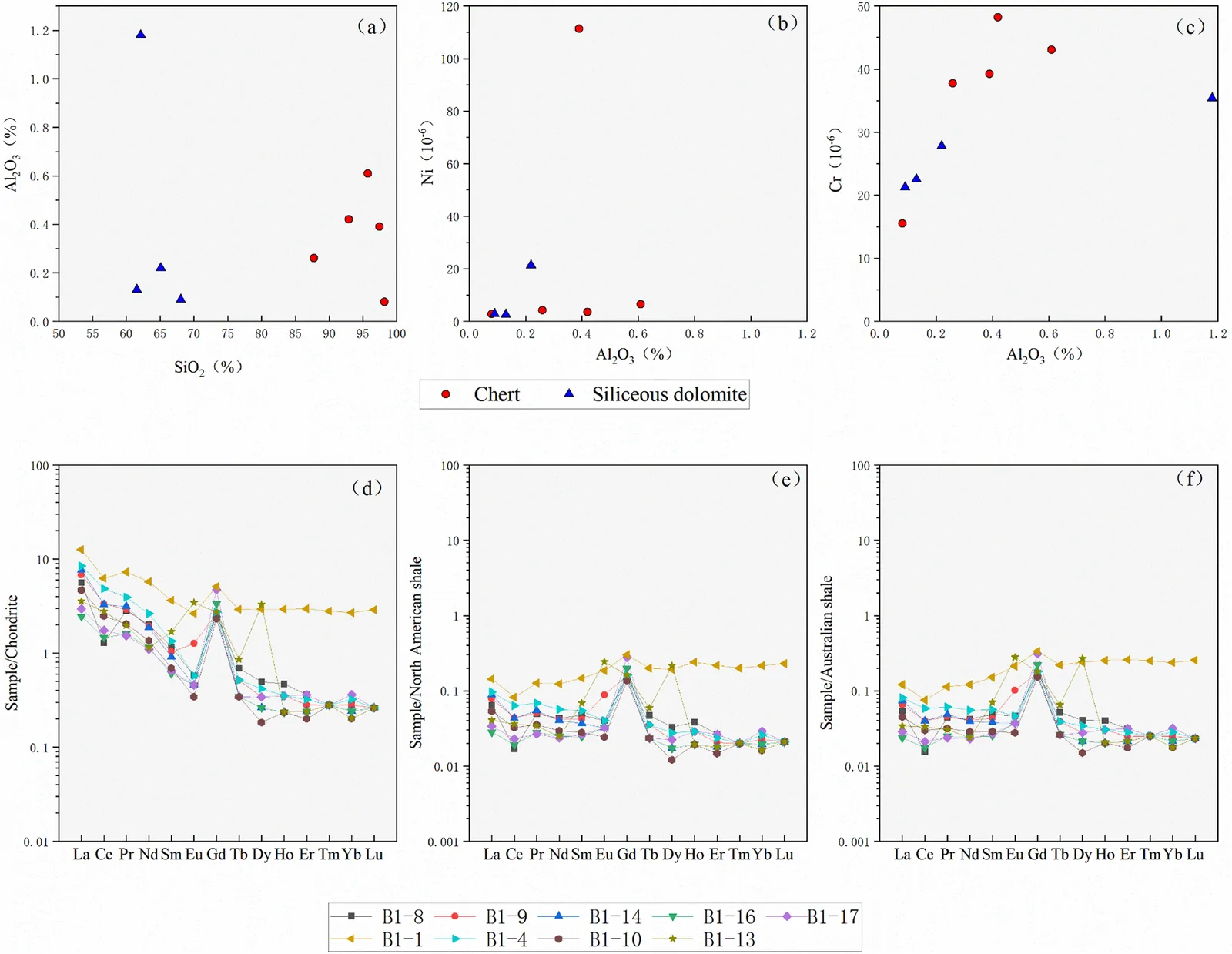
Geochemical characteristics of elements in siliceous sediments, relationships between SiO_2_ and Al_2_O_3_, as well as Al_2_O_3_ and Ni, Cr in siliceous sediments from the northern Sichuan region (a–c). Distribution patterns of rare earth element chondrite standardization (d), North American shale standardization (e), and Australian post-Archean shale standardization (f) in siliceous sediments from the northern Sichuan region.

Murray et al.^49^ showed that for hydrothermal sedimentary chert, ∑REE was less than 200 μg/g, the average value of δCe_AN_ was 0.3, which was a negative anomaly, and δEu_AN_ was a positive anomaly. The ∑REE values of the cherts and siliceous dolomites in the study area were all less than 200 μg/g, with a range of 6.0–30.0 μg/g and an average of 11.6 μg/g; the average value of δCe_AN_ was 0.7, with a range of 0.3–1.0; the average value of δEu_AN_ for the cherts and siliceous dolomites in the study area was 0.7, with a range of 0.2–2.2. Positive Eu anomaly is only observed in one siliceous dolostone sample. Combined with the tectonic background of the Ediacaran–Cambrian transition (ECT), the continuous evolution of the Deyang–Anyue rift trough and episodic crustal thinning promoted the upward migration of deep fluids. These fluids locally modified the primary seawater sedimentary system during the sedimentary and diagenetic stages, while the formation of bedded chert was predominantly controlled by deep-water biogenic sedimentation. Local physicochemical changes of seawater induced by limited fluid overprint led to silicification and metasomatism of partial microbial dolostones, forming siliceous dolostones. Meanwhile, minor siliceous fluids filled the pores of microbial dolostones during the late diagenetic stage of the Dengying Formation, recording localized, fault-controlled fluid modification under rifting background rather than pervasive hydrothermal sedimentation.

#### 5.2.3 Quantitative Contribution Analysis of Three Silica Source Endmembers

To quantitatively resolve the ambiguous coexistence of biogenic and hydrothermal signals in chert, a widely adopted three-endmember mixing model was used to discriminate the proportional contributions of biogenic silica, seawater-derived silica, and hydrothermal silica.^50,51^ Two robust proxies (Fe/Ti and Al/(Al + Fe + Mn)) with low detrital interference were applied for mass-balance fitting, and the Eu anomaly was used as an auxiliary constraint. Three classic endmembers were adopted: pure biogenic endmember (Fe/Ti = 2.0, Al/(Al + Fe + Mn) = 0.65), pure seawater endmember (Fe/Ti = 15.0, Al/(Al + Fe + Mn) = 0.40), and pure hydrothermal endmember (Fe/Ti = 60.0, Al/(Al + Fe + Mn) = 0.20).^50,51^ The fractions of the three silica sources were solved via least-squares fitting with the constraint of f1 + f2 + f3 = 1,^50,51^ ^,^ and all results were normalized to 100%.

Quantitative results demonstrate that the chert is predominantly controlled by biogenic silica (73–85%), accompanied by a stable seawater silica input (10–18%). Hydrothermal contribution is limited (4–9%) and only records localized diagenetic fluid overprint, rather than pervasive basin-scale hydrothermal deposition. Combined with low Th and Nb contents and overall negative Eu anomalies, our results clarify that the primary sedimentary origin is biogenic.

### 5.3 Evolutionary Process of the Rift Trough

Detrital zircons older than the Neoproterozoic in the study area dominantly record continental arc signatures, accompanied by arc-associated orogenic compressional events and features indicative of depleted mantle domains (Figure 5a–c). These observations are tightly linked to subduction and extensional processes along the northwestern margin of the Yangtze Block during the breakup of the Rodinia supercontinent and the assembly of Gondwana. Furthermore, crustal thickness variations calculated from Eu/Eu* ratios of detrital zircons suggest that arc-related extensional rifting initiated in the study area after ∼650 Ma.

Detrital zircon age spectra reveal populations of 1018–810 Ma and 1796–1203 Ma, which correspond to the collisional assembly stage of Rodinia. Constrained by the regional tectonic setting, the Yangtze Block constituted a fragment of Rodinia, and subduction of the Proto–Tethyan oceanic slab imparted back-arc extensional characteristics to the Deyang–Anyue zone on the northwestern Yangtze Block within an active continental margin framework (Figure 8a).^52^ At ∼605 Ma, back-arc extensional tectonics experienced tensile spreading associated with the breakup of Rodinia, which provided the dynamic mechanism for the development of the intracontinental rift and marked the onset of the Deyang–Anyue rift trough (Figure 8b).

**Figure 8:**
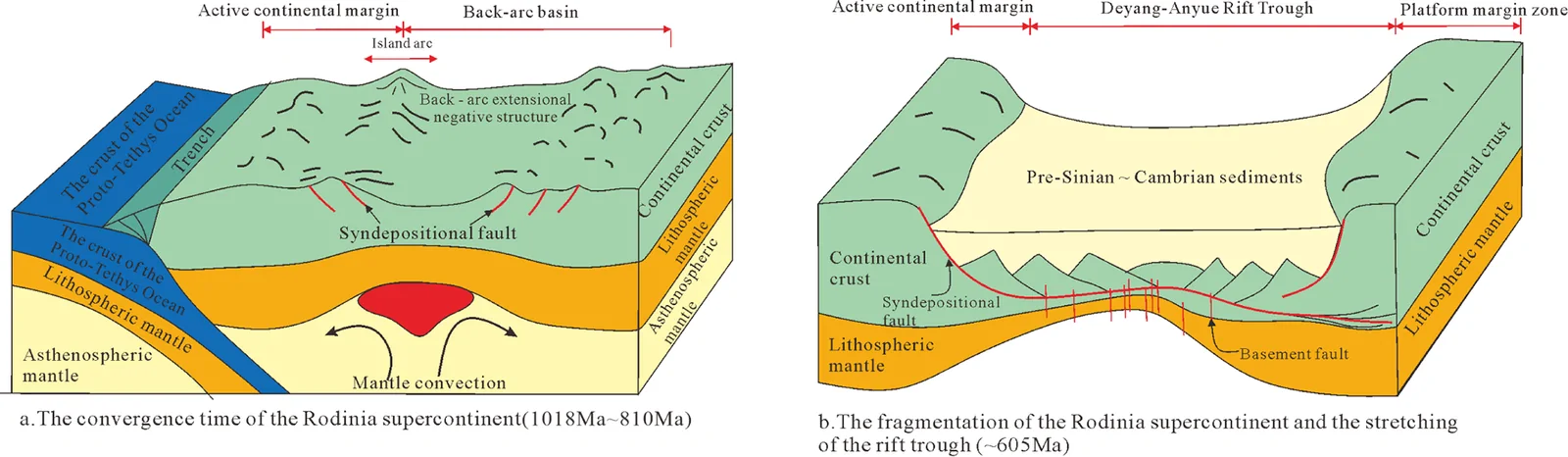
Tectonic evolution model of the rift trough on the northwestern margin of the Yangtze Platform. (a) 1018–810 Ma; (b) ∼605 Ma.

During the ECT period, the evolution of siliceous sediments is consistent with the tectonic evolutionary background of the rift trough. The Sichuan Basin was filled and deposited on the basis of the deep Nanhua period rift system. Paleotroughs developed during the depositional stage of the first and second members of the Dengying Formation and exhibited negative landforms with insignificant spatial differences (Figure 9a); the Tongwan Movement I in the late Dengying Formation experienced overall uplift, the paleotrough was deep-cut and eroded, and the second member of the Dengying Formation was thinned (Figure 9b). The third member of the Dengying Formation was transgressed during the sedimentation period, and the initial stage included muddy-water sediments with high shale contents and increased GR values, with clastic sediments at paleogeomorphic low-lying places (Figure 9c).^53–56^

**Figure 9:**
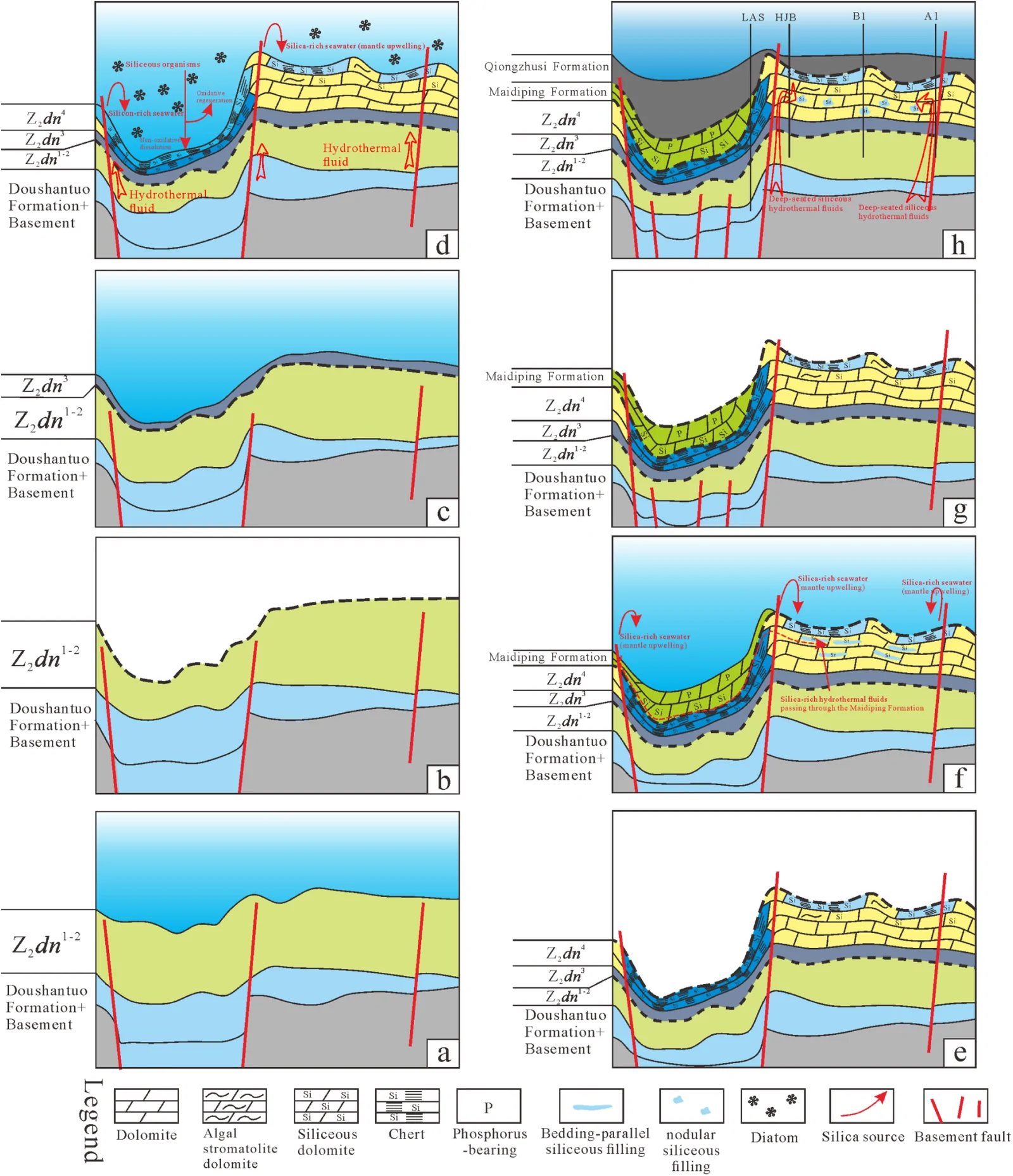
Structural evolution and peripheral siliceous evolution model of the Deyang–Anyue rift. (a) The second sedimentary period of the Dengying Formation. (b) At the end of the second stage of the Dengying Group, Act I of the Tongwan Movement. (c) The third sedimentary period of the Dengying Formation. (d) The fourth sedimentary period of the Dengying Formation. (e) At the end of the fourth stage of the Dengying Group, Act II of the Tongwan Movement. (f) The sedimentary period of the Maidiping Formation. (g) At the end of the Maidiping Formation, Act III of the Tongwan Movement. (h) Sedimentary period of the Qiongzhusi Formation.

Influenced by the movement of the Rodinia supercontinent, an obvious extensional movement in the rift trough occurred approximately 591.61 million years ago (Ma), which was the depositional interval of the Dengying Formation. During the depositional interval of the fourth member of the Dengying Formation, strong tension occurred for the first time, the deep mantle upwelled,^52^ and the silicon-rich hot seawater fluid developed in the Dengying Formation as penecontemporaneous layered chert (Figure 9d); these results indicated that the sediments were deposited in a deeper-water environment. During Episode II of the Tongwan Movement at the end of the Deng 4 Member, the Sichuan Basin was uplifted as a whole, and the top of the Deng 4 Member was exposed and eroded (Figure 9e); here, large-scale dissolution pores and caves were formed, and the shallow burial period occurred after the sea level rose again.

The second strong tension occurred during the sedimentary period of the Maidiping Formation, and the seawater was shallowed distinctly in the late depositional stage. Under the combined action of the upwelling current and continental-margin debris, collophanite and siliceous mineral sediments were deposited (Figure 9f). Internal unconformity surfaces and faults were filled with silicon-rich hydrothermal fluids and by algal dolomite of the fourth member of the Dengying Formation; at the end of the Maidiping Formation, during Episode III of the Tongwan Movement, the Sichuan Basin was uplifted as a whole (Figure 9g). During the deep-burial period of the Deng 4 Member, fractures along basement-connected faults filled the burial dissolution pores with coarse-crystalline siliceous minerals paragenetic with saddle dolomite.^57^ The trough was filled with clastic materials from the Qiongzhusi period to the late early Cambrian, and the tectonic evolution of this extensional–erosional trough ended (Figure 9h).

## 6 Conclusions

(1) Neoproterozoic zircons from the study area predominantly record continental-arc signatures, accompanied by characteristics of arc-related orogenic compression and partial contributions from depleted mantle materials. These geochemical signals are closely linked to subduction and extensional events along the northwestern margin of the Yangtze Block during the breakup of the Rodinia supercontinent and the assembly of Gondwana. Crustal thickness variations calculated from zircon Eu/Eu* ratios reveal that arc-related extensional rifting initiated in the study area after ∼650 Ma.
(2) The δ^30^Si values (relative to NBS-28) of chert range from −0.6‰ to 0.93‰, whereas siliceous dolostones yield δ^30^Si (NBS-28) values of 0.66‰to 3.26‰. Such isotopic distinctions indicate that silica in chert was derived from relatively deep-water settings with prominent biogenic contributions, while silica within siliceous dolostones originated from shallow marine environments via metasomatic processes. Trace element data demonstrate that both chert and siliceous dolostones were influenced by hydrothermal fluids during deposition.;
(3) During the ECT interval under the evolutionary framework of the rift trough, crustal thinning triggered the upward migration of deep hydrothermal fluids, which modified the seawater chemistry during sedimentation. This process promoted the accumulation of bedded chert in deep-water environments. Hydrothermally altered seawater also induced partial silicification metasomatism of microbial dolostones to form siliceous dolostones. Minor granular quartz silica fills the pore spaces of microbial dolostones during the late diagenetic stage of the Dengying Formation.

## Data Availability

The datasets supporting the findings of this study are available within the article.
